# Fasting/Refeeding Cycles Prevent Myocardial Dysfunction and Morphology Damage in the Spontaneously Hypertensive Rats

**DOI:** 10.5935/abc.20180152

**Published:** 2018-09

**Authors:** Matheus Fécchio Pinotti, Amanda Martins Matias, Mário Mateus Sugizaki, André Ferreira do Nascimento, Maeli Dal Pai, Ana Paula Lima Leopoldo, Antônio Carlos Cicogna, André Soares Leopoldo

**Affiliations:** 1 Departamento de Clínica Médica, Faculdade de Medicina, Universidade Estadual Paulista (UNESP), Botucatu, SP - Brazil; 2 Departamento de Desportos, Centro de Educação Física e Desportos, Universidade Federal do Espírito Santo (UFES), Vitória, ES - Brazil; 3 Universidade Federal de Mato Grosso (UFMT), Cuiabá, MT - Brazil; 4 Departamento de Morfologia, Instituto de Biosciências da Universidade Estadual Paulista UNESP, Botucatu, SP - Brazil

**Keywords:** Rats, Hypertension, Myocardial/dysfunction, Chronic Disease, Fasting, Reffeding, Caloric Restriction

## Abstract

**Background:**

Caloric restriction is known to impair the cardiac function and morphology in hypertrophied hearts of spontaneously hypertensive rats (SHR); however, the influence of fasting/refeeding (RF) is unknown.

**Objective:**

To investigate the fasting/refeeding approach on myocardial remodeling and function. In addition, the current study was designed to bring information regarding the mechanisms underlying the participation of Ca^2+^ handling and β-adrenergic system.

**Methods:**

Sixty-day-old male SHR rats were submitted to food *ad libitum* (C), 50% food restriction (R_50_) or RF cycles for 90 days. Cardiac remodeling was assessed by ultrastructure analysis and isolated papillary muscle function. The level of significance considered was 5% (α = 0.05).

**Results:**

The RF rats presented lower cardiac atrophy than R_50_ in relation to C rats. The C rats increased weight gain, R_50_ maintained their initial body weight and RF rats increased and decreased weight during RF. The RF did not cause functional impairment because the isotonic and isometric parameters showed similar behavior to those of C. The isotonic and isometric cardiac parameters were significantly elevated in RF rats compared to R_50_ rats. In addition, the R_50_ rats had cardiac damage in relation to C for isotonic and isometric variables. While the R_50_ rats showed focal changes in many muscle fibers, the RF rats displayed mild alterations, such as loss or disorganization of myofibrils.

**Conclusion:**

Fasting/refeeding promotes cardiac beneficial effects and attenuates myocardial injury caused by caloric restriction in SHR rats, contributing to reduce the cardiovascular risk profile and morphological injuries. Furthermore, RF promotes mild improvement in Ca^2+^ handling and β-adrenergic system.

## Introduction

The major causes of chronic non-communicable diseases (NCD)-attributable mortality are cardiovascular disease, cancers, chronic respiratory disease and diabetes.^1^ These conditions share a small number of behavioral risk factors, which aggravate the NCD and include unhealthy diet, which is closely related to hypertension.

Caloric restriction (CR) has been recognized throughout history for promoting several beneficial effects.^2,3^ Nevertheless, although CR may prevent cardiac damage in hypertrophied hearts of spontaneously hypertensive rats (SHR),^4^ it is common to note body weight fluctuations typically referred to as the "*yo-yo syndrome*" while on a regimented diet, and these fluctuations have shown deleterious cardiovascular effects.^5,6^ Researches from our laboratory and others have shown that, when dietary restriction is severe, it can promote morphological injuries and impairment of cardiac function in normal or SHR rats.^7-12^

Recently, intermittent fasting or fasting/refeeding has also shown to extend lifespan and have beneficial health effects as compared to *ad libitum* food consumption,^3,13,14^ as it enhances cardiovascular function and improves several risk factors for cardiovascular diseases.^15,16^ This dietary approach also implies a protective effect against oxidative stress, lower rates of kidney disease,^17^ prolongation of reproductive function,^18^ and leads to the normalization of resting energy expenditure and protein synthesis recuperation, but can cause many metabolic disturbances.^19,20^

In normotensive rats submitted to food restriction, chronic refeeding decreased the incidence of cardiac arrhythmia and reversed the depletion of heart proteins.^21,22^ Food restriction caused cardiac function disturbances that were almost completely reversed back to normal after chronic refeeding in the isolated rat heart.^23^ In our laboratory, we observed that fasting/refeeding cycles reversed the mechanical dysfunction and attenuated the structural injuries in papillary muscles caused by CR in normotensive rats.^24^ Nevertheless, it is not yet clear whether fasting/refeeding cycles are able to promote similar effects and/or reverse the cardiac damage induced by food restriction in SHR rats.^7-12^ Thus, the objective was to investigate the fasting/refeeding approach on myocardial remodeling and function. In addition, the current study was designed to bring information regarding the mechanisms underlying the participation of Ca^2+^ handling and β-adrenergic system. Our hypothesis is that fasting/refeeding condition would attenuate the myocardial injury caused by food restriction and would contribute to normal cardiac remodeling in SHR rats without alterations in the Ca^2+^ handling and β-adrenergic system.

## Methods

### Animal model and experimental protocol

Sixty-day-old male SHR were distributed into three groups: control (C, n = 7); food-restriction (R_50_, n = 7); and fasting/refeeding cycles (RF, n = 7). The C group was fed *Labina rat chow* containing 7.0% fat, 20.55% protein, 62.95% carbohydrate, 5.0% fiber and 4.5% moisture (Agribands, Brazil), and water was provided *ad libitum*. Table 1 shows the ingredient composition of *Labina rat chow*. The R_50_ group received 50% of the amount of food consumed by the C group. The RF group was submitted to cycles of 50% food restriction and refeeding *ad libitum* weekly. All rats were maintained on this dietary regimen for 90 days and were then euthanized.

**Table 1:** Ingredient composition of the Labina rat experimental diet

Ingredient	(g/kg)
Starch	397.5
Dextrinized Corn Starch	132.0
Sucrose	100.0
Carbohydrates	629.5
Casein	200.0
L-Cysteine	3.0
Choline bitartrate	2.5
Protein	205.5
Soy oil	70.0
Fat	70.0
Fiber	50.0
Vitamin mix	10.0
Mineral mix	35.0
Total	1000

All animals were housed in individual cages in a room maintained at 23ºC with a 12-hour light/dark cycle and were weighed once a week. Initial and final body weights (IBW and FBW, respectively), the ratios between left and right ventricular weights to final body weight (LVW/FBW and RVW/FBW, respectively) and papillary muscle cross-sectional area (CSA) were also measured. All experiments and procedures were performed in accordance with the *Guide for the Care and Use of Laboratory Animals* published by the United States National Institutes of Health and were approved by the ethics committee of Botucatu School of Medicine, UNESP, São Paulo, Brazil.

### Systolic blood pressure

Systolic blood pressure evaluation was assessed by the non-invasive tail-cuff method with a Narco BioSystems Electro-Sphygmomanometer (International Biomedical, Austin, TX, USA) at the beginning and after the end of the experimental protocol. The average of two pressure readings was recorded for each animal.

### Isolated muscle performance

Cardiac intrinsic contractile performance was evaluated by studying isolated left ventricular (LV) papillary muscle as described previously.^9,10,12^ Isometric contraction parameters, including peak of developed tension (DT, g/mm^2^, defined as peak isometric tension minus resting tension), resting tension (RT, g/mm^2^), time to peak tension (TPT, ms), peak isometric tension development rate (+dT/dt, g/mm^2^/s) and maximum tension decline rate (-dT/dt, g/mm^2^/s), time from peak tension to 50% relaxation (RT_50_, ms) were determined. The isotonic parameters were percentage of shortening (PS, %), time to peak shortening (TPS, ms), maximum shortening velocity (-dL/dt, ML/s) and maximum relaxation velocity (+dL/dt, ML/s).

The mechanical behavior of the papillary muscle was evaluated under baseline conditions at 1.25 mM [Ca^2+^] and after the following inotropic maneuvers: increase in extracellular Ca^2+^ concentration from 0.625 to 1.25, 2.5 and 5.2 mM, and β-adrenergic stimulation with 0.01, 0.1 and 1.0 *µ*M isoproterenol. The parameters used to characterize papillary muscle were as follows: length (mm), weight (mg) and CSA (mm^2^). Muscle length (ML) at peak DT was defined as L_max_ in vitro and measured with a Gartner cathetometer (Chicago, IL, USA). To compare the mechanical function between different muscle lengths, isometric and isotonic parameters were normalized to CSA and L_max_.

### Morphological study

For the ultrastructural study (three animals per group), small pieces of the LV papillary muscle were fixed in Karnovsky's fixative in 0.12 M phosphate, pH 7.2, for 1-2 hours and were postfixed in 1% osmium tetroxide in 0.1 M phosphate buffer for 2 hours.^25^ After dehydration in a graded ethanol series, the samples were embedded in epoxy resin. Ultrathin sections were cut from selected areas with a diamond knife, double-stained with uranyl acetate and lead citrate, and examined using a Philips EM 301 electron microscope. The LV myocyte CSA was measured using a compound microscope attached to a computerized imaging analysis system (Image-Pro Plus 3.0, Media Cybernetics, Silver Springs, MD, USA).

### Statistical analysis

Statistical analyses were performed using SigmaStat 3.5 software (SYSTAT Software Inc., San Jose, CA, USA). Normally distributed variables from general characteristics and myocardial function at baseline condition were reported as means ± standard deviation (SD). Comparisons between groups were performed using one-way analysis of variance (ANOVA) for independent samples. A repeated-measures two-way ANOVA was utilized to evaluate the body weight evolution and the positive and negative inotropic effects on myocardial function. When significant differences were found (p < 0.05), *post hoc* Tukey's or Bonferroni's test for multiple comparisons was carried out. The level of significance considered was 5%.

The sample size (n) was performed using the equation: n = 1 + [2C * (s/d)^2^], where C (z score α + z score β)^2^ is dependent on the values chosen for statistical power of the test (90%; type II error) and level of significance (0.05; type I error); the standard deviation value (s) adopted was 0.25, and the minimal difference between groups (d) was 0.5. The sample size needed to detect a significant difference between groups is 6.25 rats per group; however, we decided to use 7 animals per group for most of the analyses.

## Results

### General and morphological characteristics of rats

Significantly higher values of FBW, LVW, RVW, LVW/FBW and RVW/FBW were found in C compared to R_50_ and RF rats (Table 2). After 12 weeks, fasting/refeeding cycles promoted a substantial elevation of FBW and food consumption that were significantly greater than those in the R_50_ group. In relation to cardiac parameters, the RF and R_50_ groups presented different behavior. Specifically, the LVW (RF: 12.12% and R_50_: 48.5%; p < 0.05), RVW (RF: 19.04% and R_50_: 47.62%; p < 0.05), LVW/FBW (RF: 6.64% and R_50_: 19.2%; p < 0.05) and RVW/FBW (RF: 12.06% and R_50_: 18.96%; p < 0.05) were reduced in percentage in the RF and R_50_ rats as compared to C rats. Nevertheless, the fasting/refeeding cycles presented lower cardiac atrophy than R_50_ rats in relation to C rats.

**Table 2:** General characteristics of rats

Parameters	Groups		
	C	R_50_	RF
IBW (g)	247 ± 15	248 ± 12	249 ± 13
FBW (g)	366 ± 14	236 ± 17^*^	342 ± 21^*^^†^
FC (g/week)	159 ± 23	77 ± 4^*^	130 ± 55^†^
SBP initial (mmHg)	177 ± 8	177 ± 5	181 ± 7
SBP final (mmHg)	163 ± 13	157 ± 15	156 ± 5
LVW (g)	0.99 ± 0.04	0.51 ± 0.01^*^	0.87 ± 0.08^*^^†^
RVW (g)	0.21 ± 0.02	0.11 ± 0.01^*^	0.17 ± 0.02^*^^†^
LVW/FBW (mg/g)	2.71 ± 0.05	2.19 ± 0.16^*^	2.53 ± 0.08^*^^†^
RVW/FBW (mg/g)	0.58 ± 0.04	0.47 ± 0.05^*^	0.51 ± 0.05^*^

C: control group; R_50_: animals with food restriction of 50%; RF: animals with alternation between food restriction of 50% and refeeding; IBW: initial body weight; FBW: final body weight; FC: food consumption; SBP: systolic blood pressure; LVW: left ventricle weight; RVW: right ventricle weight. Values are means ± SD (n = 7).

* significant at p < 0.05 vs. C; ^†^ p < 0.05 vs. R_50_. One-way ANOVA and post hoc Tukey’s test.

**Figure 1:**
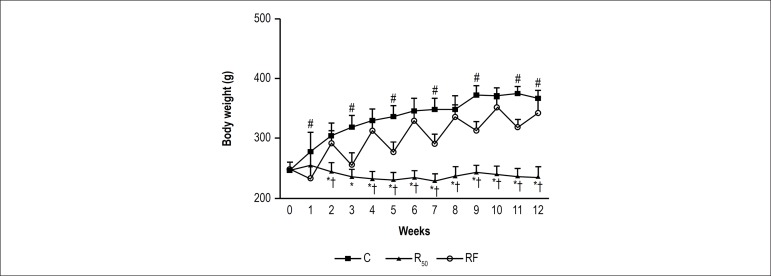
Changes in body weight after 90 days of treatment. Control (C; closed squares, n = 7), animals with food restriction of 50% (R_50_; closed triangles, n = 7) and animals with alternation between food restriction of 50% and refeeding (RF; open circles, n = 7). Values are means ± SD; * significant at p < 0.05, R_50_vs. C; ^†^ p < 0.05, RF vs. R_50_; ^#^p < 0.05, C vs. RF. Repeated measures two-way ANOVA; post hoc Bonferroni's test. Source: Research team.

In addition, C rats experienced increasing weight gain, while R_50_ rats maintained their IBW after 12 weeks of experimental protocol (Figure 1). On the other hand, RF rats gained weight dependent on food intake, with body weight increasing and decreasing during refeeding and fasting, respectively (Figure 1).

### Isolated muscle performance

Fasting/refeeding cycles did not cause functional impairment (Tables 3 and 4). Nevertheless, the isotonic [-dL/dt, TPS, and RT_50_)] and isometric parameters (TPT, +dT/dt, -dT/dt, RT_50_) were significantly elevated in RF rats compared to those in the R_50_ group, indicating that fasting/refeeding cycles preserves the contraction and relaxation phase of cardiac function. Furthermore, the R_50_ rats presented cardiac damage in relation to the C group for isotonic and isometric variables. In addition, the papillary muscle CSA showed no difference among groups.

**Table 3:** Isotonic contraction of groups at baseline condition

	Groups		
	C	R_50_	RF
PS (%)	19 ± 3	18 ± 3	20 ± 2
-dL/dt (ML/s)	1.89 ± 0.40	1.60 ± 0.36	2.19 ± 0.45^†^
TPS (ms)	168 ± 26	205 ± 14^*^	161 ± 14^†^
+dL/dt (ML/s)	4.28 ± 1.26	4.13 ± 1.11	4.79 ± 0.86
RT_50_ (ms)	58 ± 10	76 ± 12^*^	53 ± 9^†^
CSA (mm^2^)	0.95 ± 0.22	0.85 ± 0.17	0.91 ± 0.18

C: control group; R_50_: animals with food restriction of 50%; RF: animals with alternation between food restriction of 50% and refeeding; PS: percentage of shortening; -dL/dt: maximum shortening velocity; TPS: time to peak shortening; + dL/dt: maximum relaxation velocity; RT_50_: time from peak tension to 50% relaxation; CSA - muscle cross-sectional area. Values are means ± SD (n = 7) at basal calcium concentration (1.25 mM);

* significant at p < 0.05 vs. C; ^†^ p < 0.05 vs. R_50_. One‑way ANOVA and post hoc Tukey’s test

**Table 4:** Isometric contraction of groups at baseline condition

	Groups		
	C	R_50_	RF
DT (g/mm^2^)	6.17 ± 1.24	6.37 ± 1.14	7.18 ± 1.20
RT (g/mm^2^)	1.06 ± 0.12	1.12 ± 0.31	1.07 ± 0.17
+dT/dt (g/mm^2^/s)	77 ± 17	63 ± 13	93 ± 18^†^
TPT (ms)	146 ± 27	184 ± 19^*^	128 ± 25^†^
-dT/dt (g/mm^2^/s)	29 ± 5	22 ± 4	33 ± 9^†^
RT_50_ (ms)	174 ± 40	224 ± 32^*^	171 ± 21^†^
CSA (mm^2^)	0.95 ± 0.22	0.85 ± 0.17	0.91 ± 0.18

C: control group; R_50_: animals with food restriction of 50%; RF: animals with alternation between food restriction of 50% and refeeding; DT: peak developed tension; RT: resting tension; TPT: time to peak tension; +dT/dt: maximum tension development rate; -dT/dt: maximum tension decline rate; RT_50_: time from peak tension to 50% relaxation; CSA: muscle cross-sectional area. Values are means ± SD (n = 7) at basal calcium concentration (1.25 mM);

* significant at p < 0.05 vs. C; ^†^ p < 0.05 vs. R_50_. One-way ANOVA and post hoc Tukey’s test.

### Calcium stimulation

**Figure 2:**
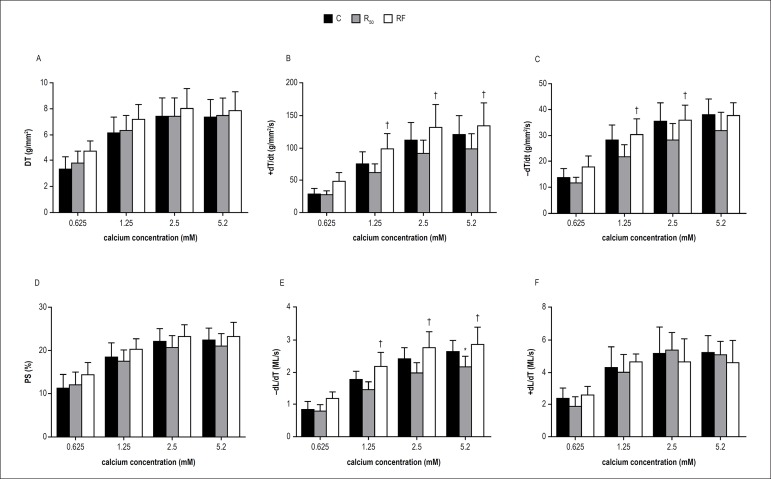
Effects of increased extracellular calcium on myocardial isotonic and isometric parameters in papillary muscles from control (C = black bars), animals with food restriction of 50% (R_50_ = gray bars) and animals with alternation between food restriction of 50% and refeeding (RF = white bars). Extracellular calcium experiment: 7 animals each group. Isometric parameters: A: DT (peak developed tension normalized per cross-sectional area); B: +dT/dt (peak isometric tension development rate normalized per cross-sectional area); C: -dT/dt, g/mm^2^/s (maximum tension decline rate normalized per cross-sectional area). Isotonic parameters: D: PS (percentage of shortening); E: -dL/dT (maximum shortening velocity); F: +dL/dT (maximum relaxation velocity). L_max_: muscle length at peak DT. Values are means ± SD; * significant at p < 0.05 vs. C; ^†^ p < 0.05 vs. R_50_. Repeated measures two-way ANOVA and post hoc Tukey's test. Source: Research team.

After baseline condition, the increases in extracellular Ca^2+^ concentrations from 0.625 to 5.2 mM resulted in a positive inotropic effect in myocytes from all groups (Figures 2A-F). However, the results shown in Figures 2B, C and E indicate that extracellular Ca^2+^ (1.25 and 2.5 mM) induced a greater response in +dT/dt (RF: 99.1 ± 23.6; 132.1 ± 36.2 g/mm^2^/s *vs.* R_50_: 63.2 ± 12.8; 91.5 ± 22.0 g/mm^2^/s; p < 0.05, respectively), -dT/dt (RF: 30.6 ± 5.9; 35.9 ± 5.8 g/mm^2^/s *vs.* R_50_: 22.0 ± 4.4; 28.5 ± 6.1 g/mm^2^/s; p < 0.05, respectively) and -dL/dt (RF: 2.19 ± 0.45; 2.77 ± 0.51 ML/s *vs.* R_50_: 1.47 ± 0.24; 1.99 ± 0.31 ML/s; p < 0.05, respectively) in the RF rats than in the R_50_ rats. In addition, -dT/dt and -dL/dt were significantly diminished in the R_50_ myocardium at Ca^2+^ concentration of 5.2 mM when compared to those in the RF group. When submitted to inotropic maneuvers, DT, PS and +dL/dt were similar between RF and R_50_. In relation to the cardiac function of C rats after Ca^2+^ stimulation, the fasting/refeeding cycles presented similar behavior (Figures 2A-F). The only significant result between C and R_50_ was noted in the highest Ca^2+^ concentration (5.2 mM); -dL/dt was significantly lower in R_50_ than in C group (C: 2.66 ± 0.35 *vs.* R_50_: 2.18 ± 0.33 ML/s, p < 0.05) (Figure 2E).

### Isoproterenol stimulation

**Figure 3:**
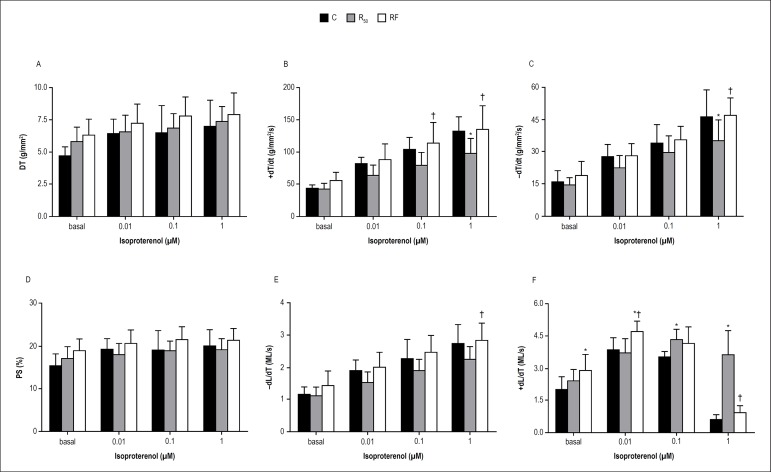
Effects of isoproterenol stimulation on myocardial function in papillary muscles from control (C = black bars), animals with food restriction of 50% (R_50_ = gray bars) and animals with alternation between food restriction of 50% and refeeding (RF = white bars). Isoproterenol stimulation experiment: 7 animals each group. Isometric parameters: A: DT (peak developed tension normalized per cross-sectional area); B: +dT/dt (peak isometric tension development rate normalized per cross-sectional area); C: -dT/dt, g/mm^2^/s (maximum tension decline rate normalized per cross-sectional area). Isotonic parameters: D: PS (percentage of shortening); E: -dL/dt (maximum shortening velocity at L_max_); F: +dL/dt (maximum relaxation velocity at L_max_). L_max_: muscle length at peak DT. Values are means ± SD; * significant at p < 0.05 vs. C; ^†^ p < 0.05 vs. R_50_.Repeated measures two-way ANOVA and post hoc Tukey's test. Source: Research team.

The fasting/refeeding cycles increased +dT/dt, -dT/dt and -dL/dt at the highest isoproterenol concentration (1 *µ*M) compared to those in the R_50_ group, indicating a positive inotropic effect in myocytes. In contrast, the RF group promoted a reduction in +dL/dt than the R_50_ group at the same isoproterenol concentration (Figure 3F). In addition, the similar effects were noted in +dT/dt and -dT/dt at 1*µ*M isoproterenol of the C group when compared to the R_50_ (Figures 3B and C). Furthermore, RF rats presented higher +dL/dt at baseline and isoproterenol concentrations (0.01 *µ*M) in comparison to C group (Figure 3F). There were no significant differences in mechanical data (DT and PS) under inotropic stimulation with isoproterenol among the groups (Figures 3A and D).

### Myocardial morphology

**Figure 4:**
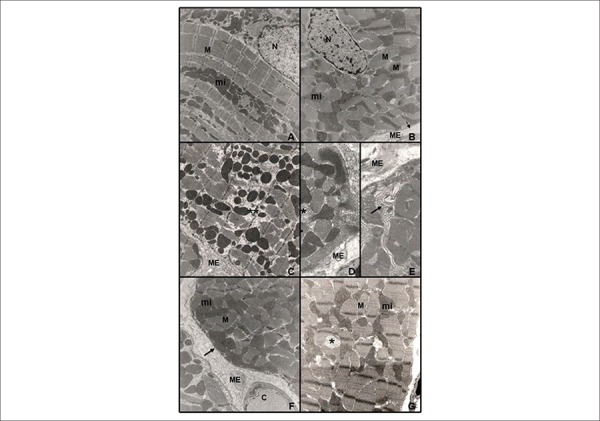
Ultrastructural study of LV papillary muscle (n = 3 per group). Photographs A and B correspond to the control group, photographs C, D and E to the food-restriction (R_50_) group and photographs F and G to the refeeding group (RF) group. The control group showed preserved ultrastructure with normal myofibrils (M), sarcoplasmic reticulum (arrowhead), mitochondria (mi), nuclear membrane (N) and plasma membrane (arrow). Food restriction rats showed cellular changes, including polymorphic mitochondria (*), myofibril disorganization (**), and infolding of the plasma membrane (arrow). The papillary muscle during refeeding showed preserved myofibrils (M), mitochondria (mi), and plasma membranes (arrow), polymorphic mitochondria (*) and capillary (C). Source: Research team.

The C group rats showed normal morphological characteristics, with myofibrils filling the entire sarcoplasm, well-defined sarcomeres, mitochondria with lamellar cristae, sarcoplasmic membranes with regular aspect, sarcoplasmic reticulum among myofibrils and nuclei with uncondensed chromatin (Figures 4A and B). The R_50_ group presented focal changes, including disorganization or absence of myofibrils, some polymorphic mitochondria with a decreased number of cristae and areas of sarcoplasmic reticulum dilation (Figures 4C, D and E). In RF rats, the only change observed was a loss of mitochondrial cristae in some organelles. Most of the fibers had normal morphology (Figures 4F and G).

## Discussion

Interestingly, little information is available on the relationship between cardiac function and morphology during fasting/refeeding in SHR hypertrophied hearts. Within this context, this dietary regimen has become the subject of considerable scientific interest for weight loss and improving cardiometabolic health. Thus, the main finding of this study was that fasting/refeeding attenuated the damage caused by CR. The results reveal that fasting/refeeding showed increased isotonic and isometric parameters at baseline, as well as improved the myocardial inotropic response to calcium and isoproterenol. In addition, fasting/refeeding prevented cardiac atrophy and morphological injuries.

Less body weight gain was observed in the RF group than in the C group (Table 1, Figure 1), but more body weight gain than in the R_50_ group. According to literature, body weight reduces approximately 13% when the animals are submitted to 48 hours of fasting.^25^ This result appears to be mediated by hormones, such as leptin that acts regulating appetite and weight gain. A rapid inhibition of *ob gene* expression in the white adipose tissue occurs in fasting, and this effect can be reversed by refeeding.^25,26^

Cardiac hypertrophy, a major pathological process involved in cardiac remodeling, initially serves as a compensatory mechanism to preserve cardiac output.^27^ Cardiac remodeling may be regarded as a first step in the sequence of adaptive responses of the heart to stress caused by a large number of physiological and pathological conditions, such as changes in volume and pressure loads and/or metabolic alterations.^28^ Current study revealed that fasting/refeeding induced cardiac atrophy visualized by reduced total heart and left ventricle, as well as in the LVW/FBW. A decrease in left ventricle weight relative to body weight is very common in small animals submitted to food restriction^22^ and fasting/refeeding.^29^ Inhibition of myocardial protein synthesis and reduction in average protein half-lives are possible explanations for reduced cardiac mass under starvation.^30^ Protein synthesis, an anabolic process, is required for cardiac hypertrophy. Two major pathways regulating protein synthesis are inhibited by AMPK, a primary regulator of metabolic pathways, which plays an essential role in a wide variety of cellular processes to protect against cardiac hypertrophy.^31^ Therefore, cardiac atrophy could be regulated by the common signaling pathway of AMPK in the hypothalamus.

In the ultrastructural analysis, food restriction caused focal morphological damage in most papillary muscle fibers. The same alterations were less intense in the intermittent refeeding condition. Intermittent refeeding seems to aid in the attenuation of the mechanisms responsible for this damage and seems to act by enhancing protein anabolism and retarding protein degradation. Recent findings suggest that the beneficial effects of refeeding result from a reduction in oxidative injury and an increase in cellular stress resistance.^2,32^ One possible mechanism for our result may be linked to the expression of atrogin-1, an E3 ubiquitin ligase also known as muscle atrophy F-box (MAFbx). E3-ligases are part of the ubiquitin proteasome pathway utilized for protein degradation during muscle atrophy. The literature has shown that atrogin-1/MAFbx expression results in muscle atrophy during catabolic condition.^33^ In cardiac muscle, atrogin-1/MAFbx expression increases during heart failure and pressure overload.^33,34^

The isolated papillary muscle analysis showed that food restriction promotes cardiac dysfunction, but refeeding condition prevents the state. These stimuli provide evidence that the improvement of myocardial function assigned to fasting/refeeding cycles was related to changes in intracellular Ca^2+^ handling, mainly in the recapture and/or extrusion of cytosolic Ca^2+^, and β-adrenergic system. Nevertheless, the lower response of food restricted rats to the increase of extracellular Ca^2+^ concentration can be related to changes in the general mechanisms involved in Ca^2+^ cycling such as sarcolemmal Na^+^/Ca^2+^ exchanger, sarcolemmal L-type channel, sarcoplasmic reticulum (SR), ryanodine receptor, SR Ca^2+^ uptake pump, and the myofilament Ca^2+^ sensitivity.^35^ In relation to RF, this process may be faster and more balanced, but no study was found to support this statement and show the activity and protein expression of Ca^2+^ handling regulatory proteins.

Another explanation could be related to the role of cytokine in intermittent fasting mediated cardioprotection. The influx of inflammatory cells and production of pro-inflammatory mediators contribute to myocardial injury.^36^ Nevertheless, adiponectin can protect myocardial cells against ischemic injury by activating the cyclic AMP-dependent protein kinase - Akt pathway, being the latter mediated, in part, by caloric restriction.^37^ Thus, the beneficial effects of fasting/refeeding may function through anti-inflammatory cytokine pathways.

Few studies have evaluated the β-adrenergic components in experimental models of fasting/refeeding.^25,35^ Some studies have shown that cardiac function impairment is related to β-adrenergic system changes,^35^ while other researchers have not reported reduced β-adrenergic response.^25^ The literature shows that a decrease in cardiac β-receptor number has been reported in several hypertensive models known to be associated with an increase in sympathetic nerve activity, including SHR.^38^ Thus, the association between increased sympathetic activity and cardiac b-receptor downregulation is sufficiently close to suggest that the finding of decreased b-receptor number after starvation and refeeding is indicative of persistently elevated cardiac sympathetic drive. However, in the current study, there is no damage of b-system in the RF rats, since the cardiac function was similar to that of the C group. The present data tend to support the hypothesis that isoproterenol stimulation reveals that the β-adrenergic system and cAMP phosphorylation of proteins related to Ca^2+^ handling were preserved in refeeding rats.

Thus, fasting/refeeding cycles have become the subject of considerable scientific interest as a potential dietary approach for weight-loss and improving cardiometabolic health. The beneficial effects of the intermittent fasting result from at least two mechanisms: the oxidative stress and the stress resistance hypothesis.^39^ According to literature, during the intermittent fasting, there are fewer free radicals produced in the mitochondria of cells and, therefore, less oxidative damage to the cells.^39^ Another hypothesis is the resistance to stress that is associated with increased resistance of cells in many different tissues to injury induced by oxidative, genotoxic and metabolic insults. The conservation of stress resistance responses to intermittent fasting across a range of species provides strong evidence that this mechanism contributes to the lifespan-extending action of dietary restriction.^39^

It is worth noting that according to studies in rodents and humans, intermittent food restriction is capable of promoting weight loss and/or favorably influence an array of cardiometabolic health indices, with equal or greater efficacy than conventional continuous energy restriction approaches, such as food restriction.^29^ Fasting/refeeding cycles increase cardiac tolerance to ischemic injury and can affect the development of cardiovascular disease, preventing postinfarct cardiac remodeling, and impending chronic heart failure.^29^ Comparing the two dietary approaches, studies show that caloric restriction may exert its beneficial effects primarily by reducing oxidative stress, whereas RF may act primarily by a stress resistance mechanism,^40^ which can have a cardioprotective effect.

### Study limitations

The study did not investigate the activity and protein expression of Ca^2+^ handling regulatory proteins known to affect myocardial contraction and relaxation. In addition, the current study did not evaluate the involvement of anti-inflammatory cytokines, free-radical production and cellular stress response, which could help and consolidate the beneficial effects of intermittent fasting.

## Conclusion

We demonstrated that fasting/refeeding promotes cardiac beneficial effects and attenuates myocardial injury caused by CR in SHR rats, contributing to the reduction of cardiovascular risk profile and morphological injuries. Furthermore, RF promotes mild improvement in the Ca^2+^ handling and β-adrenergic system.
